# The Sublethal Effects of the Entomopathic Fungus *Leptolegnia chapmanii* on Some Biological Parameters of the Dengue Vector *Aedes aegypti*

**DOI:** 10.1673/031.013.2201

**Published:** 2013-03-22

**Authors:** S.A. Pelizza, A.C. Scorsetti, M.C. Tranchida

**Affiliations:** 1 CEPAVE (Centro de Estudios Parasitólogicos y de Vectores) CCT-La Plata-CONICET-UNLP, La Plata (1900) Argentina; 2 Instituto de Botónica Carlos Spegazzini, Facultad de Ciencias Naturales y Museo, Universidad Nacional de La Plata (1900), Argentina

**Keywords:** biocontrol, dengue, fecundity, fertility, gonotrophic cycles, mosquitoes, zoospores

## Abstract

The mosquito *Aedes aegypti* (L.) (Diptera: Culicidae) is the primary vector of dengue in the Americas. The use of chemical insecticides is recommended during outbreaks of dengue in order to reduce the number of adult mosquitoes; however, because *Ae. aegypti* is highly synanthropic, the use of insecticides in densely populated areas is a dangerous practice. *Leptolegnia chapmanii* Seymour (Straminipila: Peronosporomycetes) is an entomopathogenic microorganism that has demonstrated marked pathogenicity toward the larvae of a number of mosquito species, with little or no effect on non-target insects. Therefore, the purpose of this study was to determine the sublethal effects of *L. chapmanii* on fecundity, number of gonotrophic cycles, fertility, and relationship between wing length and fecundity in *Ae. aegypti* females. *Ae. aegypti* females that survived infection with *L. chapmanii* laid fewer eggs, had a smaller number of gonotrophic cycles, had shorter wings, and were less fertile than controls. This is the first study on the sublethal effects experienced by specimens of *Ae. aegypti* that survived infection with zoospores of *L. chapmanii*. Although field studies should be carried out, the results obtained in this study are encouraging because the high and rapid larval mortality caused by *L. chapmanii* coupled with the reduction of reproductive capacity in *Ae. aegypti* females seem to cause a significant reduction in the number of adults in the mid and long term, thereby reducing the health risks associated with *Ae. aegypti*.

## Introduction

The mosquito *Aedes aegypti* (L.) (Diptera: Culicidae), widely distributed in tropical and subtropical regions, is highly adapted to urban environments. It is frequently found inside or near houses and plays an important role in the transmission of arboviruses such as dengue and urban yellow fever (Belinato et al. 2009).

Currently, control of *Ae. aegypti* is attained mainly with chemical larvicides that target the insect's central nervous system. However, the long history of insecticide use has led to the development of resistant populations all over the world. Because of this resistance, the study of novel tools to control *Ae. aegypti* and other insects of medical importance is a major area of interest (Zaim and Guillet 2002).

The use of natural enemies to control mosquitoes, based mainly on the use of products based on *Bacillus thuringiensis* var. *israelensis* (Bti), has a successful track record (Ochoa et al. 2009). Formulations of Bti are effective against larvae of *Ae. aegypti*, but its use is limited because of its high cost and low residual power, requiring regular applications of the product with the consequent increase in the cost of control programs.

Several chemical larvicides and mosquito control agents have been shown to manifest delayed effects at sublethal doses in surviving mosquitos (Simsek et al. 2009). In laboratory studies, Adugelo-Silva and Spielman (1984) have shown that inefficient larvicide reduces larval competition among survivors and increases the density and average body size of the resulting adult population. Hare and Nasci (1986) noted delayed mortality in surviving larvae of *Ae. aegypti* exposed to a median lethal concentration of Bti. Mulla and Singh (1991) examined some biological parameters and morphogenetic aberrations of *Culex quinquefasciatus* Say larvae, pupae, and adults after treating larvae with sublethal concentrations of Bti.

Recent studies have demonstrated the potential of entomopathogenic fungi to control mosquito vectors (Farenhorst et al. 2008; Mnyone et al. 2009). These fungi do not cause instant mortality, but cause sublethal and later-life lethal effects on different stages of the mosquito life cycle. Due to such properties, fungi can be potentially used as “evolutionproof agents and overcome mosquito resistance, unlike the currently deployed fastacting chemical insecticides (Mnyone et al. 2011).

*Leptolegnia chapmanii* Seymour (Straminipila: Peronosporomycetes) has demonstrated marked pathogenicity toward the larvae of a number of mosquito species, with little or no effect on non-target insects (López Lastra et al. 2004). It is important to note that until 2005, *L. chapmanii* was considered an aquatic fungi belonging to Phylum Oomycota.

In the past few years, the strain LPSC 1099ARSEF 5499 of *L. chapmanii* has shown encouraging results with regard to its pathogenicity on *Ae. aegypti*. In previous works, we studied biotic and abiotic factors affecting the infection with *L. chapmanii* (Pelizza et al. 2007a, b), and also deepened the understanding of longevity and infectivity of zoospores (Pelizza et al. 2008), the production of oogonia and oospores at different temperatures (Pelizza et al. 2010), and the combination of *L. chapmanii* zoospores with other larvicides such as Bti and temephos (Pelizza et al. 2010).

Therefore, the aim of this study was to determine the sublethal effects of *L. chapmanii* on fecundity, the number of gonotrophic cycles, fertility, and the relationship between wing length and fecundity in *Ae. aegypti* females.

## Materials and Methods

### Mosquito larvae

The *Ae. aegypti* larvae used in this study were obtained from colonies maintained following standard mosquito-rearing techniques (Gerbergetal. 1994).

### Pathogen culture

The *L. chapmanii* strain used in this study (LPSC 1099-ARSEF 5499) was obtained from a puddle with infected larvae of *Ochlerotatus albifasciatus* (Macquart) in the city of Melchor Romero, La Plata, Buenos Aires province, Argentina (López Lastra et al. 1999). The *L. chapmanii* strain LPSC 1099ARSEF 5499 was maintained on Emerson's YpSS agar medium (yeast extract 4 g, HK2PO4 1 g, MgSO4 0.5 g, starch 15 g, agar 20 g, distilled water 1000 mL) in 60 × 15 mm sterilized Petri dishes. The zoospore inoculum was obtained as previously by Pelizza et al. (2011).

### Number of gonotrophic cycles

To study the possible sublethal effects of *L. chapmanii* on *Ae. aegypti* females that survived infection with zoospores of *L. chapmanii*, 200 healthy *Ae. aegypti* larvae in the third and fourth stages and a dose of 2.2 × 10^6^ zoospores/mL of *L. chapmanii* were placed in five 1000-mL plastic containers with 800 mL of distilled water, as in a previous experiment by Pelizza et al. (2008). Additionally, 200 healthy larvae of *Ae. aegypti* in the third and fourth stages were placed in five plastic containers like to those described above and used as controls. Treated and control containers were placed in incubators at 25° C and with a 12:12 L:D photoperiod. At 24 hr, the containers were examined and pupae were placed individually in glass tubes (3.5 cm in diameter × 7.5 cm in height) with a top covered with wire mesh. Then, 4 mL of distilled water was placed in each tube and a raisin was placed on the mesh as a source of carbohydrates for adults. Treated and control pupae were maintained at 27 ± 1° C, 80% relative humidity, and 12:12 L:D. A total of 200 treated insect (100 females and 100 males) and 200 control insect (100 females and 100 males) were placed in different cages covered with a wire net (50 × 50 × 50 cm) for 72 hr, which allowed the male and female mosquitoes to mate. After this time, an immobilized hen was placed in each cage for 1 hr as a source of blood for mosquito females. After 73 hr, fed females were placed individually in glass tubes with wet paper placed in the inner perimeter to allow the females to lay eggs on the moist paper.

Treated and control females were then kept at 27 ± 1° C, 50% relative humidity, and 12:12 L:D. After oviposition, between 72 and 96 hr after ingestion of blood, eggs were counted and treated and control females that survived were returned to their respective cages. This procedure was repeated until all *Ae. aegypti* females (both treated and controls) died, and these data were used to evaluate the number of gonotrophic cycles, defined as the period between feeding the female, egg laying, and the next feeding. The experiments described above were replicated three times on different dates under similar laboratory conditions.

The numbers of eggs laid by treated and control females in each gonotrophic cycle were compared by one-way ANOVA test (raw data after checking for homogeneity of variances and normality).

**Figure 1.**
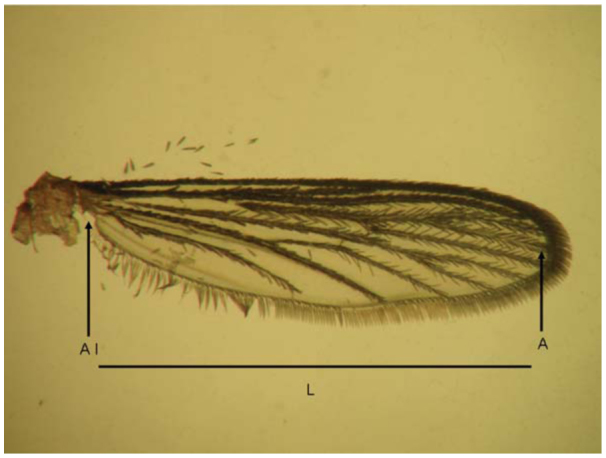
Wing of *Aedes aegypti* female. (Al) axillary incision; (A) apex; (L) maximum length. High quality figures are available online.

### Relationship between fecundity and wing length

Forewings were measured in dead females (treated and control), following the method of Packer and Corbet (1989). Both wings were measured, and the larger measurement was used for the analysis. The maximum length was determined from the axillary incision to the apex of the wing, excluding the marginal fringe (Figure 1). The experiments described above were replicated three times on different dates under similar laboratory conditions.

The wing length of treated and control females were compared by one-way ANOVA test (raw data after checking for homogeneity of variances and normality).

### Fertility

To investigate possible sublethal effects on fertility (number of viable eggs), moist papers with eggs laid in each gonotrophic cycle by both treated and control females of *Ae. aegypti* were immersed in different plastic trays (38 × 28 cm) with 4000 mL of dechlorinated water and finely ground fish as food. Trays containing the eggs laid by treated and control females were maintained at 27 ± 1° C and 12:12 L:D. First-stage larvae were counted counted and removed daily for five days. The experiments described above were replicated three times on different dates under similar laboratory conditions.

The number of viable eggs laid by treated and control females was analyzed by one-way ANOVA test and transformed by log(x+1) function to achieve homogeneity and normality.

## Results and Discussion

Significant differences were observed in the fecundity of females of *Ae. aegypti* exposed to zoospores of *L. chapmanii* compared to the controls (Table 1). Significant differences were also observed in the number of gonotrophic cycles of females of *Ae. aegypti* that survived *L. chapmanii* infection as compared to the controls (six cycles vs. eight cycles; Table 1). Significant differences (ANOVA, F = 110.90, df = 599, *p* < 0.0001) were also observed in the maximum length of the wing (1.96 mm ± 0.59 in *Ae. aegypti* females that had been in contact with *L. chapmanii* zoospores vs. 2.32 ± 0.18 mm in controls).

Significant differences were observed in fertility between females of *Ae. aegypti* exposed to zoospores of *L. chapmanii* and the controls (Table 2). The total percentage of viable eggs laid by treated *Ae. aegypti* females in their six gonotrophic cycles was 41.76 ± 5.95 %, whereas the percentage of viable eggs laid by control females was 70.23 ± 6.79 % in theireight gonotrophic cycles.

**Table 1.**
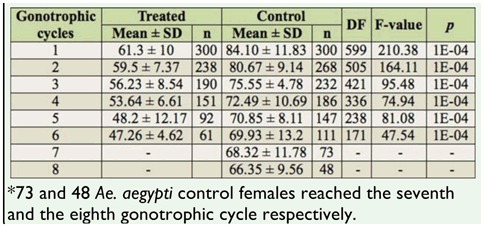
Results of the ANOVA and the average number of eggs laid by treated and control *Aedes aegypti* females for each gonotrophic cycle.

**Table 2.**
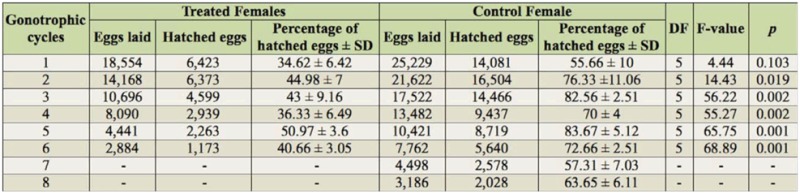
Results of the ANOVA and the percentage of hatched eggs laid by treated and control *Aedes aegypti* females in each gonotrophic cycle.

At the time of this study, the known world distribution of *L. chapmanii* is restricted to three states of the USA (California, Florida, and Ohio) and the city of Melchor Romero, La Plata, Buenos Aires province, Argentina (Mc Innis and Zattau 1982; Seymour 1984; Lord and Fukuda 1988; Fukuda et al. 1997; López Lastra et al. 1999).

This study is the first to examine the sublethal effects of *L. chapmanii* on females of *Ae. aegypti* that survived infection with *L. chapmanii*. Females that survived infection with *L. chapmanii* zoospores had smaller forewings in length and laid fewer eggs than the controls. The results are coincident with those observed by Packer and Corbet (1989) in *Aedespunctor* (Kirby) and Blakmore et al. (2000) in *Aedes albopictus* (Skuse). This study observed the correlation between body size, wing size, and the number of eggs laid, and found that females with smaller wings and smaller body size had lower fertility.

Similar results were observed by Flores et al. (2004), who applied a sublethal concentration of Bti on *Ae. aegypti* and found a significant reduction in fecundity in females of this mosquito. Also, it has been shown that three pyrethroids (d-phenothrin, d-allethrin, and tetramethrin) reduce the fecundity of *Ae. aegypti* when applied in sublethal doses (Shaalan et al. 2005). Scholte et al. (2006) observed a fecundity reduction in samples of *Anopheles gambiae* Giles treated with the entomopathogenic fungus *Metarhizium anisopliae*.

It is important to note that we observed reduced survival and therefore a reduced number of gonotrophic cycles in *Ae. aegypti* females exposed to zoospores of *L. chapmanii*. Similar results were obtained by Flores et al. (2004), who treated *Ae. aegypti* with a sublethal dose (CL70) of Bti. Mnyone et al. (2011) observed a significant reduction in survival of *An. gambiae* treated with the entomopathogenic fungi *Beauveria bassiana* and *M. anisopliae*. We observed a significant reduction in fertility (number of viable eggs) of females of *Ae. aegypti* that survived infection with zoospores of *L. chapmanii* when compared with control females. Using different sublethal doses of Bti (CL30, CL50, and CL70), Flores et al. (2004) obtained similar results, i.e., a greater reduction in survival, fecundity, and fertility in *Ae. aegypti* females when increasing the dose of this biocontrol agent. Belinato et al. (2009) evaluated *Ae. aegypti* larvae with a sublethal dose of triflumuron, a chitin synthesis inhibitor, and observed a reduction in fertility in surviving mosquito females when compared with control females.

This study marks the first time that the effects of *L. chapmanii* have been determined on *Ae. aegypti* larvae surviving *L. chapmanii* zoospore infection. This information is relevant because the decrease in survival of treated females leads to a smaller number of gonotrophic cycles. Reduced fecundity, body size (smaller length of forewings), and fertility (number of viable eggs) were also observed in treated females. Therefore, *L. chapmanii* not only causes a high and quick mortality in *Ae. aegypti* larvae, as shown in previous studies (Pelizza et al. 2008), but also reduces the reproductive capacity of larvae that survive the infection.

Although it is important to perform field studies to evaluate *L. chapmanii* as a control agent more fully, the characteristics of this control agent (high mortality and reduced reproductive capacity) seem to create a significant reduction in the population of *Ae. aegypti* in the mid and long term, and consequently reduce the health risks caused by *Ae. aegypti* as a vector of diseases.
